# Peer relations and motivation in special secondary education: Experiences of adolescents with social, emotional and behavioural difficulties

**DOI:** 10.1111/bjep.12773

**Published:** 2025-03-31

**Authors:** Willeke Norder, Anke de Boer, Alexander Minnaert

**Affiliations:** ^1^ RENN4 (Regional Expertise Centre for the North of the Netherlands for Cluster 4 Education) Groningen The Netherlands; ^2^ Department of Inclusive and Special Needs Education, Faculty of Behavioural and Social Sciences University of Groningen Groningen The Netherlands

**Keywords:** adolescents, motivation, peer relations, Self‐Determination Theory, social, emotional and behavioural difficulties, special secondary education, student experiences

## Abstract

**Background:**

According to Self‐Determination Theory (SDT), motivation is inherently present in every individual, growing from amotivation via controlled to autonomous motivation, through fulfilment of the basic psychological needs for autonomy, competence and relatedness. Peer relatedness has been found to influence motivation multimodally. Students with social, emotional and behavioural difficulties (SEBD) in special education risk additional challenges in motivation and peer relations. How peer relations influence school motivation according to SEBD students' experiences remains unclear.

**Aims:**

Investigating how peer relations influence SEBD students' motivation in special secondary education.

**Sample:**

Forty‐nine SEBD students (age 12–18) from 11 special secondary schools in the north of the Netherlands.

**Methods:**

Transcripts of semi‐structured interviews from an SDT perspective were thematically analysed for particular information on peer relations and motivation.

**Results:**

Students positively graded their motivation. Peer relations and students' motivation seemed to influence each other: (1) friends and classmates were salient reasons for school attendance; (2) peer relations influenced schoolwork motivation through relatedness, distraction, cooperation, engaging each other and being alone/loneliness.

**Conclusions:**

Consistent with previous findings, peer relations influence students' motivation in special secondary education. This influence seems reciprocal, additionally shaped by SEBD and a special education context. Feelings of (not) belonging in special education influenced school attendance and schoolwork motivation through different mechanisms, with which variations in motivational orientation, diagnosis and gender might interfere. Future research should investigate the influence of type of diagnosis, additional roles of competence and autonomy for SEBD students and possible generalizations of findings in other special and mainstream education settings.

## INTRODUCTION

Self‐Determination Theory (SDT) presents motivation as inherently present in every individual, greatly varying over time and across situations, and growing from amotivation via controlled to autonomous motivation through fulfilment of the basic psychological needs for autonomy, competence and relatedness (Deci & Ryan, 2008). When these needs are thwarted, it can lead not only to amotivation, but also to mental health challenges and psychopathology (Deci & Ryan, 2008).

Examples of mental health challenges include emotional and behavioural difficulties (EBD) (WHO, 2021). EBD, also referred to as social EBD (SEBD) (Cole et al., 2013a), are linked to psychiatric diagnoses such as ADHD (attention deficit hyperactivity disorder), CD (conduct disorder), ODD (oppositional defiant disorder) (Cole et al., 2013a, 2013b) and ASD (autism spectrum disorder) (Pandolfi & Magyar, 2014). In educational contexts, SEBD refers to ‘behavioral, social/interpersonal, and academic problems that pose formidable challenges to school personnel’ (Gable et al., 2012, p. 171). In the Netherlands, the context of the current study, students with SEBD enrol in special education when mainstream schools cannot meet their needs (as indicated by individual education plans); a multi‐track education system exists, separating mainstream and special education. Currently, approximately 4% of Dutch adolescents attend special secondary education (OCW, 2024). Just over half of that percentage, that is, 21,344 students in 2023, are students with SEBD attending special schools with SEBD expertise (OCW, 2024).

From a motivational point of view, how to best fulfill the basic psychological needs for competence, autonomy, and relatedness in an educational setting remains an ongoing challenge. Research on fostering school motivation has been conducted in a variety of contexts (Cents‐Boonstra et al., 2021; Chen et al., 2014; Haakma et al., 2017; Haerens et al., 2015; Shea et al., 2013; Shogren et al., 2019; Stroet et al., 2015). Regarding relatedness, the particular impact of peer relations on education and motivation has been investigated (Juvonen & Knifsend, 2016), showing positive effects of positive peer relations on school motivation (see Wentzel, 2017). For instance, Ryan (2001) found that one's school peer group also influences one's intrinsic motivation (i.e., enjoyment) for school (p. 1147).

Peer relations can take many forms of positive and negative relationships (Genkin et al., 2022). In school contexts, for instance, affective and collaborative peer relations have been studied (Zander et al., 2014). Wentzel (2017) shows peer relation types can influence school motivation in different ways: through informational (‘providing goals, expectations and assistance’) and motivational supports (‘emotional caring, rewards and reinforcements and peer pressure’) (p. 590).

Students with SEBD seem more prone to amotivation reflected in motivational issues such as lower intrinsic motivation, increased school absenteeism, suspension, dropout rates (Clayborne et al., 2019; Gable et al., 2012; Krezmien et al., 2006; Loopers et al., 2024; Wood et al., 2012) and lower rates of finishing tertiary education and finding employment (Dutch Inspectorate, 2023). Moreover, individuals with ADHD or autism face challenges in peer relations (Hoza, 2007; Hoza et al., 2005; Orsmond et al., 2004), and students with SEBD experience more bullying (Brown Hajdukova et al., 2016; Cho et al., 2009; Taylor et al., 2010) and, in mainstream education, have less peer relations (Pinto et al., 2019) that could benefit their motivation. Lastly, Dutch special schools have a regional function, often further away from where students live, reducing peer relation options in one's neighbourhood and causing a concentration of adolescents with SEBD in these schools.

Past research suggests within‐person differences in interactions between peer relations and motivation for students with SEBD (Loopers et al., 2024). Literature on personal experiences of SEBD students (especially in special education) that might improve understanding of such findings seems rare: despite some studies investigating SEBD students' experiences (e.g., Brown Hajdukova et al., 2016; Cassar & Abela, 2023; De Leeuw et al., 2018), such research remains limited (cf. Cefai & Cooper, 2010; Cook‐Sather, 2018; Nind et al., 2012). Considering (1) the importance of peer relations in motivation, (2) the motivational challenges within students with SEBD, (3) the disadvantages in (possibilities for developing) peer relations for SEBD students in general and within Dutch special education, (4) the underrepresentation of SEBD students' voices, studying their experiences is salient. This study therefore explores the subjectively experienced impact (further on indicated by ‘influence’/‘shape’/‘affect’) of peer relations on motivation among special secondary school students with SEBD, leading to the research question: how do peer relations, according to students with SEBD themselves, influence the motivation of these students in Dutch special secondary education? Findings will contribute to a more inclusive body of knowledge regarding motivation and to future directions for improving student motivation.

## MATERIALS AND METHODS

### Participants and procedures

To investigate the voice of students with SEBD, this qualitative study used semi‐structured interviews. Forty‐nine special secondary school students formed a maximum variation sample in terms of school location, gender, age, educational trajectory, grade and SEBD type (internalizing, such as autism, anxiety, depression and externalizing, such as ADHD, CD and ODD). All students were enrolled in classes of a maximum 14 students in one of 11 special secondary school locations. All schools were part of a special education organization in the Northern provinces of the Netherlands (Drenthe, Groningen, Friesland). Table 1 provides an overview of participant characteristics.

**TABLE 1:** Participant characteristics.

Total sample: 49			
Age		Educational trajectory^a^	
12	4	Arbeid/PRO	11
13	6	BB	3
14	19	BB/KB	6
15	10	KB	7
16	5	TL	14
17	4	TL/HAVO	3
18	1	HAVO	5

Gender^b^		Grade	
Male	34	Year 1	10
Female	15	Year 2	14
		Year 3	17
		Year 4	8

^a^ Arbeid/PRO (*Praktijkonderwijs*), BB (*Basisberoepsgerichte Leerweg*), KB (*Kaderberoepsgerichte Leerweg*), TL (*Theoretische Leerweg*), HAVO (*Hoger Algemeen Voortgezet Onderwijs*). The first is a practical education. BB, KB and TL are all 4‐year routes for pre‐vocational secondary education (VMBO), BB being the most practical and TL being the most theoretical. All lead to a different levels of secondary vocational education. HAVO is senior general secondary education, leading to tertiary higher education. When two educational levels are mentioned, the student was following education in a group with both levels, or the definite level had not been determined yet by the school at the time of the interview. The schools provide several levels, resulting in a mixed population. Students in all levels receive theoretical as well as practical classes.

^b^ This gender division is representative of the overall gender division in the included secondary special education schools.

Interviews were conducted from August 2022 to April 2023 at students' school locations. Consent procedures, interviews and transcriptions were performed by a doctoral researcher and two master students in pedagogical sciences specializing in special education. All students in the schools were eligible for inclusion, except those whose well‐being could be adversely influenced by participation or those no longer attending school awaiting placement at another institution. Informed consent for participating in and audio recording of the interview, and access to the schools' digital personal student file with background information was required for participation. For students younger than 16, parental/guardian informed consent was required additionally.

To invite students to participate, schools' behavioural scientists became contact persons and invited students through teachers.^1^ In some instances, students were approached specifically to reach the maximum variation sample. Interviews took place during students' regular school hours. Interview moments were arranged with the student and their teacher to fit timetables and not to miss any favourite classes (e.g., sports or internship activities).

The ethical committee of the University of Groningen, The Netherlands, provided ethical approval for the study (reference: PED‐2122‐S‐0078). According to the committee's ethical regulations, all data were stored on the university web drive and are only accessible to the research team and the research director.

### Measures

Interviews were conducted as part of a larger study on motivation and well‐being in special education from an SDT perspective. Interviews were semi‐structured and lasted approximately 30 min to just over 1 hr. Interviewers were trained in using a guideline with six coloured themes based on SDT and possible questions per theme (Table 2). Theme cards in corresponding colours were placed on the table to structure the interview for the students. After discussing a topic, cards were turned upside down by the interviewer or student. An additional card with a self‐rating scale of 1–10, corresponding with the Dutch grading system, could be used to clarify answers.

**TABLE 2:** Interview guideline.

Theme	Main questions
1. Narrative (‘Your story’)	Can you tell me how you arrived here at this school?
2. Well‐being (‘Feeling at school’)	How do you like it at your school? How do you feel at school? Do you feel like going to school in the mornings?
3. Motivation (‘Motivation’)	How motivated are you for school? How would you grade your motivation for school, and why? Why do you go to school? Would you go to school if it would not be mandatory? What do you like most at school? What do you find most important at school? Why do you think school is important?
4. Competence (‘Talents’)	Do you have the feeling you are going forward at school? When you have the feeling you are learning something new, how does that feel? When do you feel you are learning? Do you/your classmates, friends, or teachers, do something so that you become better at things? (How) does the above influence your motivation?
5. Relatedness (‘Friends, classmates and teachers’)	Do you feel at home at school, and why? Do you have friends and nice classmates at school? How is your relationship with teachers? Do you ever feel lonely or alone at school? (How) does the above influence your motivation?
6. Autonomy (‘Making choices’)	How do you like to make your own choices? When are moments you can choose something in your own way at school? What are things at school you would want to be able to decide for yourself? What happens if you could make choices about (too) many things at school? (How) does the above influence your motivation?

Theme names in brackets are the theme names as were visible for the students. The fragments that were coded for the current study (see Table 3) mainly came from Categories 2, 3 and 5. Interviews were semi‐structured. The example questions in this table were used to elicit more detailed responses.

**TABLE 3:** Codebook for the current study.

Theme	Category	Code
Motivation	Motivation in general	Student definitions
		Grade, feeling and description
	Determinants for going to school^a^	Fellow students (peers, friends, classmates)
	Determinants for doing schoolwork^a^	Fellow students (peers, friends, classmates)
Relatedness	General feeling of relatedness	Loneliness
		Description current general feeling of relatedness^b^, ^c^
	Peers, friends, classmates, fellow students	Description current feeling of relatedness with fellow students^c^

^a^ The codes ‘determinants for going to school’ and ‘determinants for doing schoolwork” fell into a broader category in the larger study, and thus, IRR, and included *all* determinants for motivation to go to school and to do schoolwork, respectively. For the current study, this code was used only when peer relations were the determinant for school attendance and/or schoolwork motivation.

^b^ The code ‘description current feeling of relatedness’ was added after the IRR and therefore not included in the IRR as such. It had first been part of a general code for well‐being experiences.

^c^ The codes ‘description current feeling at school’ and ‘description current feeling of relatedness with fellow students’ were used only as background information for the current study.

### Data analysis

Transcripts of audio recorded interviews were entered into Atlas.ti 23. Based on coding five pilot interviews, the researcher made a preliminary codebook for the whole data set of the larger study. Data were analysed following thematic analysis (Braun & Clarke, 2006). This process included deductive coding (broad coding based on the SDT concepts) and inductive coding (detailed coding based on what students shared about their experiences in their personal narratives and within the concepts of motivation, well‐being, competence, autonomy and relatedness) after thorough familiarization with the data (cf. Braun & Clarke, 2006, p. 87).

The preliminary codebook for the whole study was tested and discussed extensively within the research team, and then condensed and clarified. IRR for the whole dataset was calculated. As some IRR values were below .67, intensive reflection was undertaken within the research team and in consultation with an expert in the field. This resulted in consensus about the codebook, and in agreement that the interconnectedness between the basic psychological needs might deflate the IRR. For the current study, only a subset of codes was applicable for analysis (Table 3). Calculating the IRR for this subset led to a decreased IRR (Krippendorf's Alpha Binary was .55) due to a smaller number of codes to be included. However, the researchers assume this is not a reliable IRR, since some adaptations (see notes with Table 3) could not be included while they were made after conducting and critically reflecting on the IRR process. The researchers therefore concluded that obtaining IRR for this sub study was challenging through calculations, yet agreed that sufficient consensus was reached through careful reflections and considerations within the research team.

Following thematic analysis (Braun & Clarke, 2006), the fragments coded with the codes in Table 3 were reread, searching for themes. Thematic maps were drafted manually to eventually define the themes that helped structure the line for this paper.

## RESULTS

### Student definitions of motivation

Students described their understanding of the concept of motivation. Some students knew the word, yet did not know how to describe it in their own words. Many definitions included the idea of feeling like doing something or liking to do something. Some students described motivation as a feeling from within or a reason for doing something. Several definitions included the will or urge to do something, or the energy or effort to put into something one does or has to do. Some students gave definitions in which they also shared the importance of having a goal. As one boy explained:

That reminds me of a game which you probably don't know. Hmm…that you're focused on a goal, and that you don't 2 feel like wanting to give up. (30‐M‐12) 3

Several students brought up the concept of concentration when talking about motivation. Considering that, students distinguished wanting to do something and being able to do something:

Motivation is wanting to work; concentration is being able to work. (21‐M‐17)

[Motivation is] a little like how much you might feel like doing something. (… ) And also, how much energy you want to put into something, or how much energy you can put into something. Bit of a combination. Sometimes that is that you want to do it, but that you just can't. (32‐M‐18)

A few students mentioned mindset and strategies in defining motivation, such as discipline, perseverance, trying your best, and being positive:

That you think of something that makes you feel like doing it and that you really want to do it. (10‐F‐14)

That you can turn yourself on, so to speak, to learn for your work and that at school you just do your best to work. (19‐F‐15)

### General experiences of motivation

The average grade 32 students assigned to their motivation on the self‐rating scale (1–10) was 6.3. Grades ranged between 3 and 8.75. Six students rated their motivation with a grade under 5.5, equalizing an insufficient mark in the Dutch grading system. Students were asked to explain how it feels to be motivated. One student said:

That basically means that I just, just feel good, and that I am just doing my work well, and then I think: okay, it's good. (9‐M‐14)

When asked whether they felt like going to school in the mornings, many students stated that when waking up they felt poorly motivated for school (because of tiredness, sleepiness or having to take the taxi or bicycle to get to school); yet, once at school they would usually feel alright. One student stated this was not a surprise because he is an adolescent who just likes to sleep. Another student explained:

I wake up in the morning, and then I think about why I woke up and then I'm like: ‘Oh yeah, I have to go to school’. And then … yeah, I go to school, and then I become happy when I'm at school. (14‐F‐14)

A few students were explicitly positive about their motivation. One 13‐year‐old boy explained he even wanted to go to school on days off, as he liked it so much. Others were explicitly negative about their motivation. For instance, one girl, after grading her motivation with 6.5, stated:

Usually, I don't really feel like school. (…) Yeah, I just don't feel like it because then I'm looking forward to fun things. (…) And school isn't fun. (24‐F‐12)

Another student said:

I don't think I'll be motivated at this school. [The last day before the holidays,] I see something different, I guess. A lot (…) more colour, or something. Lighter, or whatever. [And when the holidays are almost over, I feel,] just, well, like crap. (42‐M‐16)

Many students shared their coping strategies for having to go to school each day even though they might not feel like it. They put the obligation to be at school into perspective by explaining it is normal for students not to always like school; by stating there are good and bad things at school; and by saying that school is ‘just’ school.

On the one hand I don't feel like it, and on the other hand I do. I do think everyone has that. There are fun aspects to school, but there are also annoying ones. (12‐M‐14)

Yeah, it's not fun, sometimes. But it isn't like every day, you hate it, you don't want to, you're not going (…) I mean, (…) you can't say no, basically. (41‐M‐15)

### Peers influencing school motivation

Students explained how their peers shaped their motivation for school. Their stories distinguish between the object of motivation when talking about peers; peers influence the motivation to attend school and, in different ways, the motivation to do schoolwork once at school. Both are presented in this section.

### Peers influencing school attendance motivation

#### Friends and classmates are reasons to go to school

The majority of students mentioned peers as one of the most important reasons to go to school. Multiple students indicated that because of their friends and classmates, they felt like going to school more, they experienced more joy in school, they felt happier going to school or they thought school was nice. A few students stated that the most important reason to go to school were friends, not being alone, classmates whom they had a good relationship with and social contact. Some students indicated that the most important reasons for them to keep liking school was the family feeling, enjoying time with friends, being nice with each other or having friends at school. One student was asked which things he really did for himself when going to school. One of his answers were ‘friends’. Some boys stated:

How ‘gezellig’^4^ it is with the class, (…) that's usually my biggest motivator to go to school, so, I kind of think that that is the nicest [thing] about school. (21‐M‐17)

If I would have been the only one here at school, I would probably not even show up anymore. (6‐M‐17)

Some students indicated that on days or mornings with low motivation, knowing they would meet peers again at school would motivate them to go. Also, when imagining school was not mandatory, several students mentioned they would still go to school, given they would meet their friends and classmates there. Some of them emphasized that this point of view had been influenced by earlier episodes of school dropout where they had missed their peers while being at home for weeks or months. One boy's response when asked whether he would go to school if it was not mandatory illustrated the importance of peer relations in going to school:

I feel like other people in my class wouldn't do it, but I, I personally would (…) if the whole class was there, then I would [go]. If I were on my own or whatever, then it's, it's not really fun. (40‐M‐15)

A few students presented the importance of learning social skills that would help them in later life as a reason to go to school even if it would not be mandatory. For instance:

School, as a school‐situation, is something you can't really recreate. (…) Once you leave school, you can't really experience it ever again. (…) it's something I've always felt apprehensive about, but it is, it is something you mentally need, I think, because everyone has it, so to speak. (…) if you've never been to school, then I think it would be very weird, because the rest of the people did go to school; school is a bit of an experience; everyone should have experienced it. (2‐M‐15)

Some students indicated that they would not go to school, or that they would go to school less if it were not mandatory, in order to be able to spend more time with their friends outside school.

Practical suggestions to use the help of peers to get to school came up in speaking about cycling to school together with a peer. However, students nuanced such appointments did not always guarantee motivation to go to school, and that life or just liking to stay at home could still get in the way of actually going to school.

A few students explained how (earlier) bullying at school or not having friends or nice classmates affected their feeling of enjoying school. Such situations were not automatically linked to low motivation, although one girl explained that she felt slightly anxious to go to school in the morning because she was afraid her peers would bully her.

Within their stories, students distinguished friends from classmates when talking about peer relations and motivation. Several times, students stated it was not about classmates, but about friends. Others mentioned friends and fellow students separately when talking about the matter. One student explained that the influence of both groups could be quite different when he was asked whether he felt like going to school in the mornings:

To see friends and stuff, yeah. Having fun, laughing, seeing friends again, playing together, playing games, but there's also a part of you that goes: ‘I should really get on with the kids I don't (…) necessarily like’. But then you think of your friends again, and then you're like: actually, actually I do like that. (26‐M‐13)

A few students indicated that friends were not really a motivation for them to go to school, yet more of a by‐effect. As one girl indicated when she was asked what was most important for learning:

Friends, I think, those don't really matter that much to me personally. I think that school is more fun because of it, but it's not exactly made for it. (19‐F‐15)

For several students, their SEBD diagnosis or challenges in terms of mood or oppositional behaviour could interfere with how peer relations shaped their motivation for going to school. A few students shared that when not feeling well mentally, despite a general ‘gezellig’ feeling at school, arguments and fights with peers could result, which would make them want to leave early or play truant the following day. Positive influences of mood on peer relations and motivation also existed:

Sometimes I feel extra comfortable in my own skin, and then I'm really happy to go to school. Just to see everyone again, to go do things. (…) At moments like those I do [rate my motivation a nine out of ten]. (20‐M‐12)

### Peers influencing schoolwork motivation

Multiple students said they did not believe their peers really influenced their motivation to do schoolwork. As this 14‐year‐old girl stated:

I think [my motivation for school subjects] remains the same, because (…) other kids don't have that much to say about your subjects. (1‐F‐14)

Further inquiry on peer interactions in the classroom showed that peers can influence one's schoolwork motivation through five factors: feelings of relatedness, distractions in the classroom, cooperation, engaging each other and loneliness/being alone.

#### Relatedness

Experiences of (un)relatedness in special education influenced the motivation to do schoolwork. Several students spoke about feeling ‘too normal’ for special education, considering the SEBD of their peers more extreme than their own. Experiences around relatedness could both increase and decrease student motivation. For example:

I don't come here to make friends, or to be friendly with people … I just come here to go to school. Do what I have to, and then leave again, just, as soon as possible. (…) I think that if you (…) don't feel at home, that it can have an impact, like: I don't belong here, I don't want to do my best, you know? These are not the people I want to be with. But, on the other hand, it does sometimes motivate too, like: I don't belong here, so I want to get out of here as quickly as possible. (23‐M‐15)

Since I don't feel well here (…) I'm immediately, like: “Yeah, I'll, uhm, take a step back.” (…) Just the fact that I'm in special education. Look, maybe it's normal for others, but I feel too special here, so to speak. (…)

Interviewer: Is there something (…) that does motivate you greatly at school, despite all these things?

Yeah, just fighting to be able to go to a different school. (39‐F‐13)

#### Distractions in the classroom

Distractions in the classroom, mentioned in approximately half of the interviews, were often said to reduce one's concentration, motivation and the extent to which students liked being in the classroom. Many students emphasized preferring a quiet classroom to be motivated and do their work. One female student indicated she had the least motivation when her class was busy. Another girl explained:

I am always motivated, but sometimes the kids in my class just really demotivate me. If everyone's busy, and not as in, occupied with the test, but just busy in general, then I can't do my test correctly either. (49‐F‐15)

Distractions were explained to be due to classmates' talking and a ‘busy classroom’, considered something that could be positive as well as negative. Examples of positive sides included that talking with each other is considered ‘*gezellig’*, and that it is not boring in the classroom with laughter and jokes. This positive side was indicated to also be able to become negative in the form of distraction, for instance when things got ‘too *gezellig’* in the classroom (13‐M‐16).

Students presented the following consequences of a ‘busy classroom’: feeling powerless, annoyed, tired; becoming more hyperactive themselves than usually with their SEBD; not being able to continue working. Some students shared strategies of sitting with other, quieter, classmates or of listening to music through headphones to avoid being distracted by classmates.

A few students said that the influence of negative or disruptive behaviour also depends on who would distract them. Several students indicated that distractions were not only caused by classmates, but also partly by themselves, for instance, due to lack of motivation. In these wordings, students honestly opened up about their own responsibility in this:

Usually, I'm the one making the most noise in class. So it might be better if I'm the one that has to be quiet, and the others are basically doing quite alright. The others are just quiet. (…) When I'm not quiet, I'm not motivated to work, and then I try to stir up other people, (…) and then I'm not going to be seriously doing my work. (5‐M‐14)

And I do it myself, I myself take part in it as well, it's not like: classmates [are] bad, hahaha. I take part in it myself, all that talking and moving about. I also like that. And yeah, sometimes, sometimes that's a little too distracting for others. (13‐M‐16)

Other forms of distractions in the classroom included oppositional classmate behaviour such as fights, verbal aggression, or disturbing behaviour of classmates resulting from their SEBD diagnosis. A few students stated they did not feel disrupted by negative behaviour of others, and they would easily start it themselves. One girl indicated she often started distraction by being rude to her classmates, yet being in another classroom with older students helped her not to show negative behaviour, and increased her motivation. Some students explained how distraction by negative peer behaviour as part of peers' SEBD could prompt them to also engage in negative behaviour. A few reflected on how they tried to avoid this:

I [am] also not really interested in (…) them [i.e., classmates]. (…). They're the wrong crowd for me. And some [of them] really don't interest me either. (…) They're very impulsive. I'm like that too, for sure, but when I'm not around impulsive people, I'm less impulsive myself as well. (40‐M‐15)

In our class (…): a few more rude children, which I'm one off myself as well sometimes. (…) calling each other names, ridiculing each other. (…) I'm kind of used to it by now, but, yeah, when I used to see that in the past, I'm like: ‘Ok, what is this?’ (…) Also, at certain times I think: ‘Okay, where did I end up!?’ (…) then people are acting really weird, or whatever, and then I'm like: ‘Okay … ?’ Every now and then I participate in that as well and then I'm like: ‘Yeah.’ (…) When I'm properly motivated, I don't participate, when I'm (…), a bit further away from my work, then I mostly participate. (9‐M‐14)

#### Cooperation

Cooperation between students was not as common in the curriculum for all students. Some of them indicated this is due to different grades and educational routes in their group. Others explained that during practical classes, cooperation was more common. Several students were explicitly positive about (optional) cooperation with classmates. Explanations for positive attitudes included: importance of cooperating skills for the future; liking cooperation or finding it nice; contributing to concentration or to motivation; helping each other; learning from each other:

I do sometimes do economics together with a classmate. And, uhm, that motivates me as well, because, yeah, he can explain it pretty well, and in the meantime (…) we can also just have nice conversations. Because we are, essentially, good friends, so then it usually turns into a bit of working and chatting, and that motivates me as well. (7‐F‐17)

I think that [working together] can give a little extra motivation (…). Because, uh, you can then also talk with each other a little, (…) you get a bit of distraction. That might make [it] a bit easier sometimes. (…) if you, (…) work on your own, so to speak, for the entire day, then you might, I think, be less motivated, or lose your concentration quicker than when you're doing something together with a [female] friend or whatever, because then you're, uhm, not, so to speak, working by yourself the whole time. (11‐F‐17)

Cooperation could be practical by making assignments together. It could also help with one's (SEBD) challenges such as dyslexia or concentrating. As one boy explained:

I think working together (…) is great, because on my own, I'm not very good at it, I have strong dyslexia as well, so I do really like working in groups. (6‐M‐17)

Students explained that whether cooperation was helpful depended on who they were collaborating with. As these students shared:

Sometimes we have to do things in groups and that kind of stuff. (…) Yeah, like: ooh, I can't with that person, or oh, I can't with that one. And the same the other way around. Then it's not going all that well. (18‐M‐12)

I try not to spend time on people with low motivation, because that may be the only thing that can bring my motivation down a little. (21‐M‐17)

One student shared cooperation would not increase schoolwork motivation, yet could help doing another activity inside the classroom. This boy with severe low motivation, low energy and depressed mood had found motivation for a creative activity together with his classmates, leading to ‘doing something, at least’ (32‐M‐18).

#### Engaging each other

Several students showed examples of how they and their classmates engaged each other. In one instance, this was a very practical motivating action:

And then I made a deal with one of my friends about homework, so now I just do homework every day. (…) we are both going to do homework daily (…) the one who gives up first before the 16th of May has to buy the other a tray of energy [drink]. (28‐F‐16)

Another girl explained she really enjoyed the arts class because only those students who really wanted to be in that class joined. One girl stated that her being motivated could also be reduced by her classmates laughing at her because of her motivation.

Another way of engaging each other was by giving compliments, for instance by saying to someone ‘you're doing it well!’ (36‐M‐13). One 15‐year‐old girl who indicated to struggle with fear of failure stated that her fellow students' compliments helped her to gain motivation.

#### Being alone and loneliness

A few students shared experiences of feeling alone or lonely at school. Although loneliness was sometimes said to slightly reduce motivation, being alone was not always experienced negatively. From time to time, students even identified being alone for a while as pleasant. Some students presented being alone as a way to regain schoolwork motivation. For instance, one boy shared that when things were not good at home, he would feel less motivated for schoolwork, and working individually outside the classroom would help him. Another student said:

In places like school I do prefer to be alone, during breaks or whatever, because then I can think. And by thinking, I can build the motivation again to (…) when I get back to the classroom again. If I would work in a book, I would be ready to handle it, so to speak. By being alone for a while, by thinking, and then …by resetting myself. So being alone is important, I feel. (2‐M‐15)

## DISCUSSION

**FIGURE 1:**
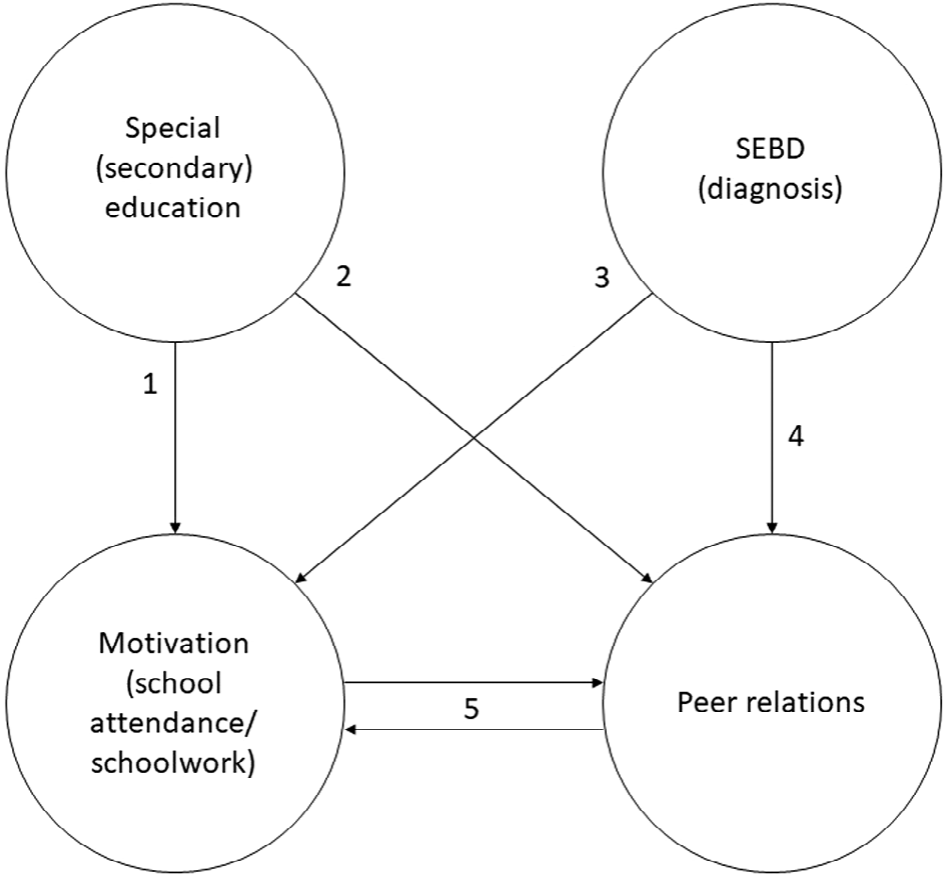
Visualization of the postulated relationships between motivation and peer relations within the context of special secondary education and SEBD. This visualization is suggested as a working model that allows for further exploration on the issue and does not claim the particular causal relationships. The postulated relationships can be understood as follows, based on the current findings: (1) Not wanting to be in special education yet in ‘normal’ education (can be because of peers, see 2; yet can also simply be because of the building, the stigma on special education or the restrictions of special education) influencing motivation. (2) Feeling ‘too normal’ for special education, or just not finding like‐minded peers, influences peer relations (and, in turn, affecting motivation). (3) (Not) feeling well due to mental health related to SEBD influencing one's motivation, and possibly in turn affecting peer relations. (4) (Not) feeling well due to mental health related to SEBD influencing whether or not and how to connect with others, in turn affecting motivation for school (e.g., not feeling well, interacting less pleasantly with peers, experiencing less motivation to stay at school or attend the next day). (5) Includes some of the examples of motivation–peer relations interactions above and the described interactions in the results section (i.e., through belonging, relatedness, distraction, engaging each other, cooperation and being alone/loneliness).

This study investigated how peer relations influence SEBD students' motivation in special secondary education, according to these students' subjective experiences. Results underline earlier findings that peer relations influence student motivation in several ways. Moreover, they suggest a bidirectional relationship: peer relations not only influence motivation; motivation also shapes peer relations in the context investigated. Additionally, this study indicates a distinction between how school attendance motivation and schoolwork motivation are influenced by and interact with peer relations. Moreover, it suggests that a context of SEBD and special education adds additional dynamics to these interactions (visualized in Figure 1).

Feelings of belonging in the special education context illustrates the diversity of possible ties in peer relations (Genkin et al., 2022). Students feeling ‘too normal’ had an attitude of ‘contempt’ (Genkin et al., 2022; one of the negative ties) towards their peers with SEBD (even though they themselves have SEBD too). Although ‘similarity between friends is (…) an important basis for the maintenance of friendships’ (Veenstra & Laninga‐Wijnen, 2022), struggling with SEBD does not seem to be the similarity that automatically brings students together. Rather, some students shared their reluctance of becoming too close with peers for fear of also developing ‘bad behaviour’ or losing motivation—a phenomenon that could be explained through the concept of ‘selection similarity’ (Veenstra & Laninga‐Wijnen, 2022). This fear seems legitimate for losing attention and showing problematic school behaviour (see Geven et al., 2013). Interestingly, some students acknowledged they could be, for example, impulsive or hyperactive themselves, yet (attempted to) choose not affiliating themselves with peers that were disruptive. This indicates autonomy and self‐reflection—which became clear from students' narratives—as well as self‐regulation skills. Some students identified how their personal motivation mediated their strength of resisting ‘bad influences’. This suggests (a more autonomous) motivation can be both an outcome of and a mediator for peer relationships; a phenomenon also identified by Shao et al. (2024).

The average grade students gave their school attendance motivation was above passing grade, contrary to literature suggesting low motivation among students with SEBD (cf. Clayborne et al., 2019; Gable et al., 2012; Krezmien et al., 2006; Wood et al., 2012). Students often explained their passing grades for motivation referring to feelings of fun and nice people at school, indicating satisfying school peer relations can increase students' motivation, consistent with studies in mainstream education (e.g., Ryan, 2001). Considering the high dropout rates among students with SEBD (cf. Gable et al., 2012; Wood et al., 2012), this finding seems still more hopeful, since results imply satisfying peer relations may be helpful in improving school attendance. This mechanism, however, appears to work differently for autonomously motivated students (considering peers as less determining for their school motivation) than for students with more controlled school motivation (who refer to friends as main reason for school attendance and whose motivation to see friends might also be fulfilled outside of school). Interestingly, some students (especially those who experienced temporary dropout) seemed to combine these motivational orientations, connecting the utility value (cf. Ryan, 2001) of school attendance with social interactions and learning and developing social skills. This phenomenon was also presented by Wentzel (2017), stating that peers, through ‘facilitating the development of (…) social skills’ (p. 590) can interact in how peer relations shape school motivation.

Students from both groups performed autonomous self‐regulating learning strategies to navigate their way through (mandatory) school life, reflecting the need for autonomy for all students. Such strategies, mainly coming up while talking about distractions in the classroom, included isolating or withdrawing oneself from peers' noise or negatively behaving classmates: an example of attention control, which is ‘an important predictor of student academic motivation’ (Zumbrunn et al., 2011, p. 3). Strategies also related to concentration; a concept linked with motivation in this and earlier research (e.g., Bonifacci et al., 2023). Another self‐regulating learning strategy was help‐seeking (see Zumbrunn et al., 2011) from peers for schoolwork through cooperating with classmates, yet also for social skills through engaging each other. Help‐seeking can be seen as example of peers' ‘collaborative ties’ (Zander et al., 2014, p. 420), while engaging each other is an example of ‘affective ties’ in the form of emotional support (idem). The use of such ties as self‐regulating strategies is interesting in further understanding the suspected lower help‐seeking among students with SEBD, which mechanisms remain under researched (Davison et al., 2022).

Within the special education context, within‐ and between‐person variability (Martin et al., 2015; Minnaert, 2023) of diagnosis and gender require special attention. As indicated, one's diagnosis can influence students' mood and feelings, shaping motivation; how they respond to and respect peers; how peer relations influence motivation. All students had internalizing and/or externalizing SEBD, with or without formal diagnosis. Prior SDT‐based research (age 8–16) (Morsink et al., 2019) shows students with externalizing challenges need different approaches (e.g., external rewards) to boost task motivation than students with internalizing challenges (e.g., less cooperation and less distraction). This underlines the need for differentiated approaches in the classroom that also consider peer relations.

Considering gender, girls seemed to more often talk about being distracted and having negative feelings regarding peer relatedness. A possible reason for this might be their underrepresentation in Dutch secondary special education: one or two girls in a group of a maximum 14 students is common, possibly influencing girls' feelings of belonging, relatedness, and friendships, for example, as most students' friendships are same‐gender friendships (Shin & Ryan, 2014). Other research (Liem & Martin, 2011) shows positive influences of peer relations on school engagement are stronger when they are same‐sex peer relations; something to consider in Dutch special education where fewer options exist for girls to build same‐sex peer relations. Current results, unfortunately, do not provide unequivocal conclusions about gender influences on peer relations. Past research, however, identified differences in motivation orientation and self‐regulating skills between boys and girls (Driessen & Van Langen, 2010; OECD, 2015), and mechanisms through which gender interferes with peer influences on motivation (Shin & Ryan, 2014).

### Limitations

This study discusses the subjectively perceived influences of peer relations on motivation. Therefore, no objective causality claims can be made from the study's results. Second, this study is limited to students' relatedness and motivation. According to SDT, the needs for autonomy and competence additionally influence these dynamics; yet, these are not considered in the current research. Third, the IRR for this study could not be fully captured in purely quantitative measures and was therefore supplemented by thorough reflection within the research team and with an external expert. Fourth, a possible selection bias towards more positively motivated and verbally stronger students is a limitation. Furthermore, a more ethnically diverse sample would have allowed for an even more inclusive understanding. Lastly, the current study does not provide in‐depth insight into possible differences between the experiences of students with internalizing and externalizing SEBD.

## CONCLUSION

Results of this qualitative study on peer relations and motivation in special secondary education are in line with prior research in mainstream educational settings: peer relations play an important role in shaping student motivation. Even more so, this influence seems reciprocal: peer relations and motivation interact with each other in the context investigated. Furthermore, the context of SEBD and special education seems to give an additional dimension to this reciprocal interaction. Feeling to (not) belong in special education appeared to be a salient aspect in influencing motivation, especially and first of all in motivation for school attendance. Also in schoolwork motivation, for which peer relations can be experienced as practical ways and resources yet also as obstacles (through relatedness, distraction, cooperation, engaging each other, being alone/loneliness), this feeling plays a role. Variations in how important students considered peers and friends in influencing their motivation also seem to be related to their motivational orientation (i.e., controlled or more autonomous motivation). Differences in diagnosis and gender might additionally influence the interactions between peer relations and motivation in special education. The influence of such inter‐ and intraindividual differences (e.g., internalizing or externalizing SEBD) should be addressed in future research.

Although results shine a light on the subjectively experienced mechanisms of interactions between peer relations and motivation, they cannot automatically be generalized to other contexts. Future research that further investigates this topic in special as well as mainstream education is therefore needed. Additionally, SDT‐based research that also includes the basic needs for autonomy and competence is required in the special educational context for a more comprehensive understanding. Meanwhile, educational policy should already (1) consider the influence of a special education context on the dynamics between peer relations and motivation in discussions regarding inclusive education; and (2) maintain options for more custom‐made approaches for students with SEBD when working towards inclusive education. In practice, special as well as mainstream schools should keep investing in school and classroom environments fostering feelings of peer relatedness to contribute to student motivation.

## AUTHOR CONTRIBUTIONS

**Willeke Norder:** Writing – original draft; writing – review and editing; visualization; methodology; investigation; conceptualization; funding acquisition; project administration; formal analysis; data curation; validation. **Anke de Boer:** Supervision; writing – review and editing; conceptualization; methodology; validation. **Alexander Minnaert:** Supervision; writing – review and editing; conceptualization; methodology; validation.

## CONFLICT OF INTEREST STATEMENT

The authors declare they have no conflict of interest.

## Data Availability

The data supporting the findings of this study are available from the corresponding author upon motivated request.

## Footnotes

^1^
All grade groups have one or two teachers (*mentor*) who 9 teach(es) the entire day and knows the students best. This teacher generally teaches all subjects, although additional teachers for practical lessons and vocational training (e.g., cooking, technical skills, sports, art), and, sporadically, some theoretical subjects, are present in the schools too.

^2^
Emphasis in expression indicated as underlined text.

^3^
(Participant number‐gender‐age). All expressions are translated from Dutch by the principle investigator and a native English speaker.

^4^
The word ‘gezellig’ is difficult to translate. Students often used it to talk about their experiences in the class. It refers to a nice, pleasant, sociable and happy vibe or fun with others in the classroom.
